# The depletion of gut microbiome impairs the beneficial effect of Gui-Shen-Wan in restoring mice ovarian function and associated protein expression of ovarian tissues

**DOI:** 10.3389/fcimb.2024.1505958

**Published:** 2024-11-27

**Authors:** Xingtao Huang, Ruinan Xu, Qin Yang, Xin Jiang, Jinju Lin, Huashan Zhao, Ruifang Wu, Hui Du, Wenkui Dai

**Affiliations:** ^1^ Department of Traditional Chinese Medicine, Peking University Shenzhen Hospital, Shenzhen, China; ^2^ Department of Obstetrics and Gynecology, Peking University Shenzhen Hospital, Shenzhen, China; ^3^ Institute of Obstetrics and Gynecology, Shenzhen Peking University-Hong Kong University of Science and Technology (PKU-HKUST) Medical Center, Shenzhen, China; ^4^ Shenzhen Key Laboratory on Technology for Early Diagnosis of Major Gynecologic Diseases, Shenzhen, China; ^5^ Center for Energy Metabolism and Reproduction, Shenzhen Institutes of Advanced Technology, Chinese Academy of Sciences, Shenzhen, China

**Keywords:** traditional Chinese medicine, Gui-Shen-Wan, gut microbiome, ovarian dysfunction, ovarian proteome

## Abstract

**Introduction:**

Traditional Chinese Medicine (TCM), specifically Gui-Shen-Wan, has shown promise in restoring ovarian function among reproductive-age women who had impaired ovarian functions, yet the underlying mechanisms remain elusive. Recent studies highlight the pivotal role of the gut microbiome (GM) in mediating the therapeutic effects of TCM. However, it is unclear whether the GM contributes to Gui-Shen-Wan’s therapeutic restoration of ovarian functions.

**Methods:**

This study employed a mouse model with cyclophosphamide-induced decreased ovarian function (P_T and P_AT groups) and a control group without modeling. The P_AT group received a 7-day course of oral antibiotics to deplete the GM prior to a 20-day Gui-Shen-Wan treatment regimen.

**Results:**

Both P_T and P_AT mice exhibited prolonged metestrus/diestrus phases compared to controls (p<0.05), indicating menstrual disruption post-modeling. Following 20 days of Gui-Shen-Wan treatment, P_T mice showed a shorter metestrus/diestrus phase (4 days) compared to P_AT mice (5 days) (p<0.05). Notably, P_T mice had a higher number of normal follicles(primitive/primary/secondary/antral follicles) in their ovaries post-treatment (median 15) compared to P_AT mice (median 8.5). Proteome analysis revealed that ovarian proteins enriched in P_T mice were primarily associated with oxidative phosphorylation and DNA replication pathways, suggesting GM-mediated enhancement of these processes.

**Discussion:**

This study underscores the pivotal role of the GM in the therapeutic benefits of Gui-Shen-Wan, highlighting the potential for microbiome-targeted interventions in promoting beneficial effects of Gui-Shen-Wan on the restoration of decreased ovarian functions.

## Introduction

Decreased ovarian function represents a significant challenge to fertility in a substantial proportion of reproductive-age women globally (Carson and Kallen, 2021). Hormone replacement therapy (HRT), while has been widely employed to maintain menstrual regularity and alleviate symptoms associated with low estrogen levels (Committee on Gynecologic Practice, 2017). Nevertheless, HRT has limitations in replicating a fully functional ovary and is associated with adverse effects, including an elevated risk of cardiovascular diseases, over the long term (Donnez and Dolmans, 2018; Agarwal et al., 2018). Consequently, there is a pressing need for alternative therapeutic approaches.

Recent research has highlighted the potential of Traditional Chinese Medicine (TCM) in mitigating premature ovarian insufficiency (POI) and restoring ovarian function, offering promising alternatives to conventional HRT (Peng, 2015; Zhou et al., 2022; Tan et al., 2018; Duan and Cheng, 2021). Despite these promising outcomes, the underlying mechanisms mediating the therapeutic effects of TCM remain largely unknown.

Emerging evidence underscores the pivotal role of gut microbiome (GM) in modulating the efficacy of TCM therapies (Zou et al., 2022; Lee et al., 2020; Gou et al., 2023; Li et al., 2023; Liu et al., 2023; Chen et al., 2023; Ammar et al., 2023). Zou Y et al. demonstrated the involvement of GM in the nephroprotective effects of the TCM formula Qiong-Yu-Gao against cisplatin-induced acute kidney injury (AKI) (Zou et al., 2022), while another study revealed the GM-mediated therapeutic effects of Yijin-Tang on hepatosteatosis and insulin resistance (Lee et al., 2020). Similar GM-mediated mechanisms have been documented for various TCM formulations, including Pien Tze Huang, Quercetin, Xiaoyaosan, and Arula-7 powder (Gou et al., 2023; Li et al., 2023; Liu et al., 2023; Chen et al., 2023). Furthermore, GM can directly metabolize components within TCM formulas, thereby contributing to their beneficial effects (Ammar et al., 2023).

Given the established efficacy of Gui-Shen-Wan in mitigating decreased ovarian function (Zhou et al., 2024; Tian et al., 2022), this study endeavors to investigate the potential role of GM in mediating the therapeutic effects of Gui-Shen-Wan. By shedding light on the interplay between Gui-Shen-Wan and GM, our findings will enhance the understanding of the mechanisms underlying Gui-Shen-Wan’s beneficial effects on ovarian function restoration, potentially paving the way for GM-targeted therapeutic strategies.

## Materials and methods

### Ethics statement and animal selection

**Figure 1:**
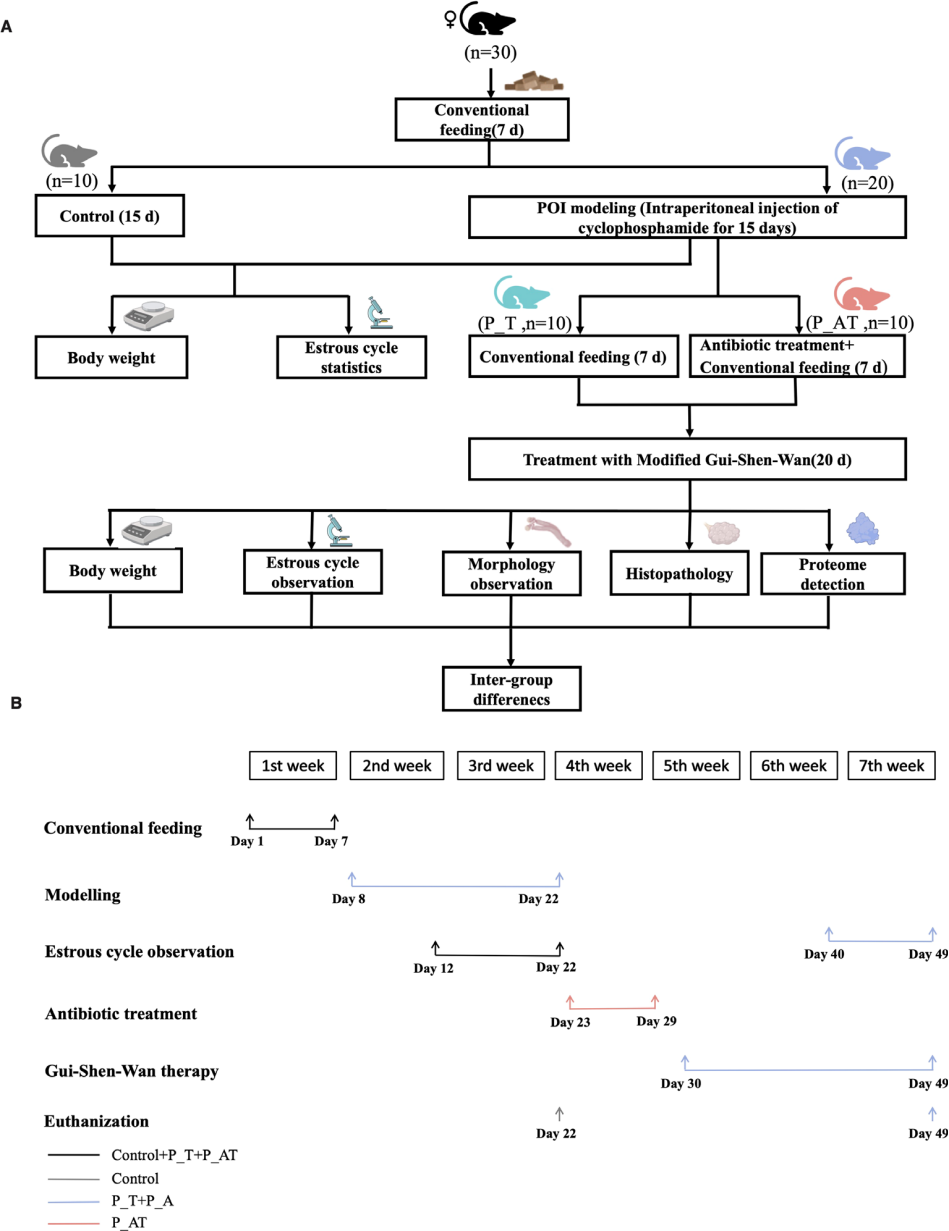
Study design. **(A)** Research pipeline. **(B)** Time-around for the animal experiment.

This study was conducted in accordance with the guidelines approved by the Institutional Animal Care and Use Committee of Shenzhen Institutes of Advanced Technology, Chinese Academy of Sciences. Female SPF C57B/6J mice aged 5 weeks (purchased from Weitonglihua, China) were selected for the establishment of a mouse model with impaired ovarian function (Mohamed et al., 2019). Prior to the experiment, mice were allowed to acclimatize for one week in a ventilated cage maintained at room temperature with a 12-hour light-dark cycle. Daily body weights were recorded at 9:00 AM to monitor animal health and well-being (**Figure 1A**).

### Experimental design and group allocation

A total of 30 mice were randomly assigned to three groups, each comprising 10 animals: (1) Control group (routine feeding without modelling), (2) P_T group (model mice receiving TCM therapy post-modelling), and (3) P_AT group (model mice subjected to GM depletion via antibiotics followed by TCM therapy post-modelling).

### Modelling of impaired ovarian function

Following established protocols (Liu et al., 2024), mice in the P_T and P_AT groups underwent intraperitoneal injection of cyclophosphamide (MCE, USA) to induce ovarian dysfunction. Specifically, on the first day of modelling, these mice received an initial dose of 100 mg/kg cyclophosphamide, followed by daily injections of 8 mg/kg for 14 consecutive days. Mice in the Control group received an equal volume of sterile phosphate-buffered saline (PBS) via intraperitoneal injection for 15 days. To assess the estrous cycle, vaginal smears were collected daily from the 6th to 15th day of modelling, at 10:00 AM. On the 15th day, mice in the Control group were euthanized.

### GM depletion

To deplete the GM in the P_AT group, mice received a cocktail of antibiotics in their drinking water from the 16th day post-modelling for 7 consecutive days. The antibiotic mixture consisted of 1 g/L metronidazole, 1 g/L neomycin sulfate, 1 g/L ampicillin sodium, and 0.5 g/L vancomycin hydrochloride, as previously reported (Liu et al., 2023; Qi et al., 2019). To ensure consistent antibiotic concentrations, the drinking water was replaced daily. Additionally, both P_T and P_AT mice received 3% sucrose in their drinking water to enhance palatability and ensure adequate hydration.

### TCM therapy and tissue collection

On the 23rd day post-modeling, P_AT mice were administered regular drinking water, mirroring the treatment of P_T mice. Subsequently, both P_T and P_AT mice underwent a 20-day regimen of TCM therapy in the form of Gui-Shen-Wan (**Figure 1B**). Gui-Shen-Wan was formulated with a combination of 10g each of Danshen tablets, Goji berries, Rehmannia glutinosa, Epimedium, Beichaihu, Roasted licorice, and Salt Dodder Seed. Notably, the drug concentration was scaled down from clinical dosages to account for the difference in body weight between humans and mice. For adult humans, the recommended daily dose of each Gui-Shen-Wan ingredient is 10g, equating to 0.0002g per gram of body weight per day for a 50-kg individual. Given the average mouse weight of 20g, the initial calculated daily dose for each mouse was 0.004g. However, to mitigate potential vomiting after gavage, the dose was adjusted upwards to 0.016g per mouse, which was dissolved in 0.2ml of sterile water for administration.

### Estrous cycle observation and pathology of ovarian tissues

From the 33rd to 42nd day post-modeling, vaginal smear examinations were performed daily at 10:00 AM to monitor the estrous cycle in both P_T and P_AT mice (**Figure 1B**). On the 42nd day, P_T and P_AT mice were euthanized for comprehensive tissue analysis. For ovarian tissue pathology, the skin and muscle layers were dissected from the abdomen to procure bilateral ovarian tissue. One ovary from each mouse was promptly fixed in 10% neutral buffered formalin and processed for paraffin embedding. Serial 5 µm tissue sections were prepared from the paraffin blocks and stained with hematoxylin and eosin (H&E) for microscopic examination under 4× and 10× magnifications. The number of primitive, primary, secondary and antral follicles present in each tissue section was quantified.

### Proteome analysis of ovarian tissues

The freshly procured ovarian tissues were promptly frozen using dry ice for a minimum of ten minutes to ensure rapid and effective preservation. Following this, the tissues were stored at -80°C for subsequent analysis. To investigate protein expression profiles within each sample, we employed Label-free Data-Independent Acquisition (DIA) technology, a highly sensitive and quantitative method for proteome analysis (Zhang et al., 2020).

The DIA-NN software(version 1.8) was utilized for protein identification. This software was configured to search the Uniprot database, applying stringent criteria (PSM FDR < 0.01 and Protein FDR < 0.01) to ensure high confidence in protein identifications (Demichev et al., 2020). The identified proteins were then subjected to functional annotation, leveraging the comprehensive resources of GO (Gene Ontology) and KEGG (Kyoto Encyclopedia of Genes and Genomes) databases, providing insights into their biological roles and pathways.

For quantitative assessment of protein abundance, we again utilized the DIA-NN software(version 1.8). This process yielded a matrix of normalized protein abundances, which served as the foundation for subsequent statistical and bioinformatic analyses. This matrix ensured that comparisons across samples were accurate and unbiased, allowing for the identification of meaningful differences in protein expression patterns.

### Statistical analysis

Inter-group differences was analyzed via wilcoxon rank-sum test. The visualization was conducted via R software (version 4.0.5).

## Results

### Study design

Thirty C57B/6J mice were subjected to routine feeding for a period of 7 days prior to experimental manipulation. Subsequently, the mice were evenly divided into three groups: Control, P_T, and P_AT, with ten mice in each group (**Figure 1**). Mice in the P_T and P_AT groups were injected with cyclophosphamide daily for 15 consecutive days, while mice in the Control group received injections of sterile PBS during this period. This experimental design aimed to induce menstrual disturbances in the P_T and P_AT groups.

Following the cyclophosphamide treatment, mice in the P_T and P_AT groups were assigned to two different feeding protocols: conventional feeding for the P_T group and conventional feeding supplemented with antibiotics for the P_AT group, both for a duration of 7 days. Following the completion of these feeding protocols and subsequent treatment with TCM for both P_T and P_AT groups, morphological evaluations of the ovaries were conducted. Furthermore, pathology and proteome analyses were performed on ovarian tissues to gain insights into the potential role of GM in the therapeutic effects of Gui-Shen-Wan.

### P_T and P_AT mice have different estrous cycle compared to mice in the control group

**Figure 2:**
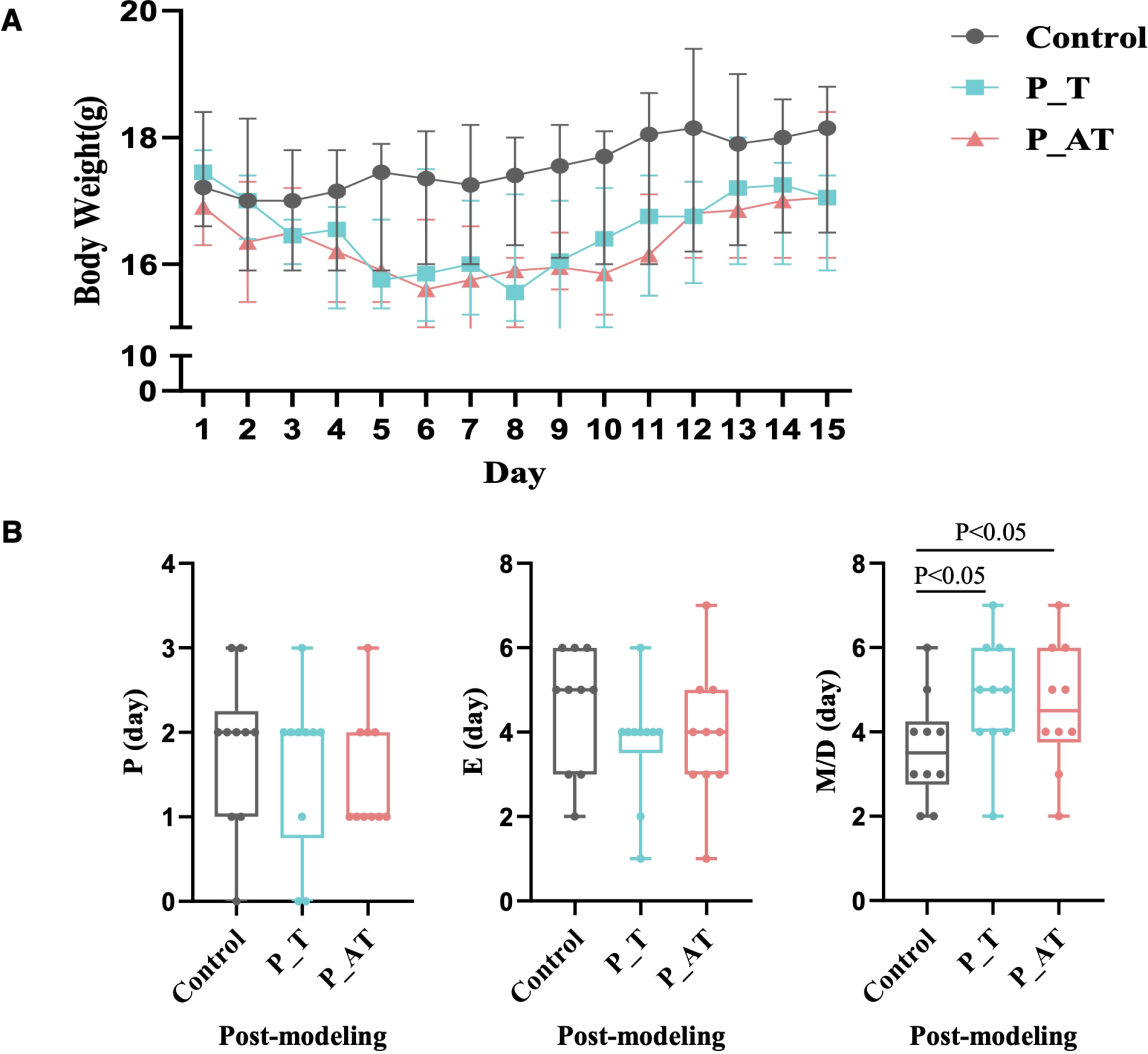
Inter-group differences of the body weight and estrous cycle. **(A)** The differences of body weight between Control, P_T and P_AT mice (n=10 for each group). The central point represents median. The upper and lower line represent upper quartile and lower quartile, respectively. **(B)** The differences of estrous cycle between Control, P_T and P_AT mice. The central line represents median. P, proestrus; E, estrus; M, metestrus; D, diestrus.

Significant alterations in body weight were observed in the P_T and P_AT groups compared to the Control group (**Figure 2A**). Specifically, on the fifth day of modeling, both P_T and P_AT mice exhibited an average weight loss of 7.9%. This trend persisted from the 6th to 10th day of modeling, with the average weights of P_T and P_AT mice remaining lower than those of Control mice (15.97g and 15.81g versus 17.45g, respectively). Even at the end of the 15-day modeling period, the weights of P_T and P_AT mice remained approximately 6% lower than those of Control mice (**Figure 2A**).

Additionally, variations in the estrous cycle were notable among three groups. Compared to the Control group, mice in the P_T and P_AT groups exhibited shorter proestrus (P) and estrus (E) phases, accompanied by notably longer metestrus (M)/diestrus (D) phases. Specifically, the median M/D duration was found to be 42.8% longer in P_T mice (5 days versus 3.5 days in the Control group) and 28.6% longer in P_AT mice (4.5 days versus 3.5 days in the Control group), with statistical significance (p<0.05) observed for both comparisons (**Figure 2B**).

### There are significant post-therapy differences of estrous cycle and ovarian pathology between P_T and P_AT mice

After 7-day exposure to antibiotics, a notable decrease in bod weight was observed in P_AT mice compared to P_T mice (**Supplementary Figure S1**). However, during the period of TCM therapy, no statistically significant difference in body weight was detected between the P_T and P_AT groups (**Supplementary Figure S1**).

**Figure 3:**
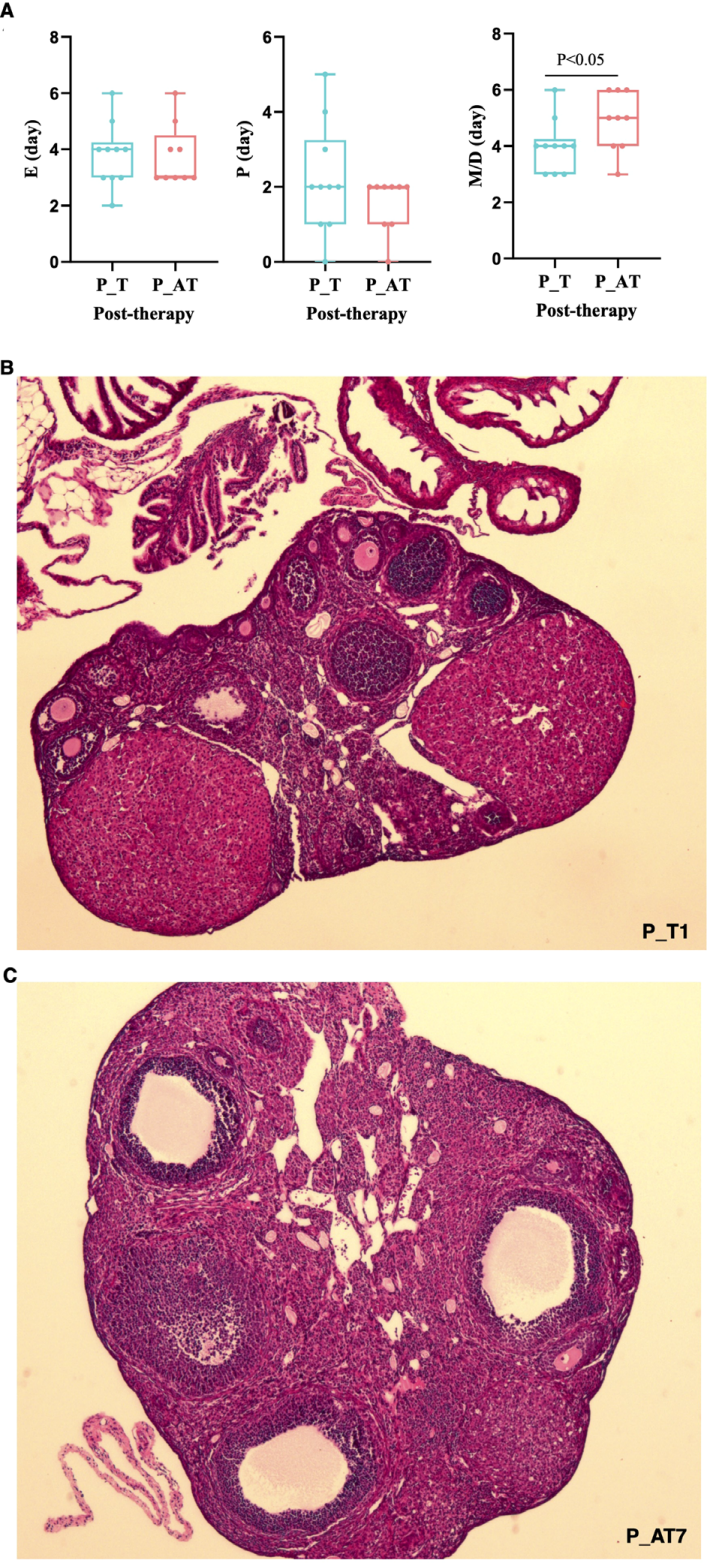
Differences of therapeutic effects between P_T and P_AT mice. **(A)** Distribution of proestrus(P), estrus(E), metestrus/diestrus(M/D) for P_T(n=10) and P_AT(n=9) mice. The central line represents median. **(B, C)** Histology pathology of ovarian tissues for P_T and P_AT mice under 4× field view.

Despite lacking statistical significance, the P (proestrus) and E (estrus) phases of the estrous cycle in P_AT mice were shorter than those in P_T mice following therapy (**Figure 3A**). Conversely, the M/D (metestrus/diestrus) phase of the estrous cycle was significantly longer in P_AT mice, with an average duration of 5 days compared to 4 days in P_T mice (p<0.05).

Hematoxylin and Eosin (H&E) staining revealed a pronounced reduction in the number of normal follicles(primitive/primary/secondary/antral follicles) in P_AT mice (median: 8.5, range: 4 to 15) when compared to P_T mice (median: 15, range: 7 to 19) (**Figures 3B, C**; **Supplementary Table S1**). Both at estrus phase in the estrous cycle, mouse P_T1 exhibited 19 normal follicles within a 4× microscopic field of view, whereas mouse P_AT7 had only 4 normal follicles under the same conditions (**Figures 3B, C**).

### Protein expression of ovary tissues in P_T mice notably differed from that in P_AT mice

**Figure 4:**
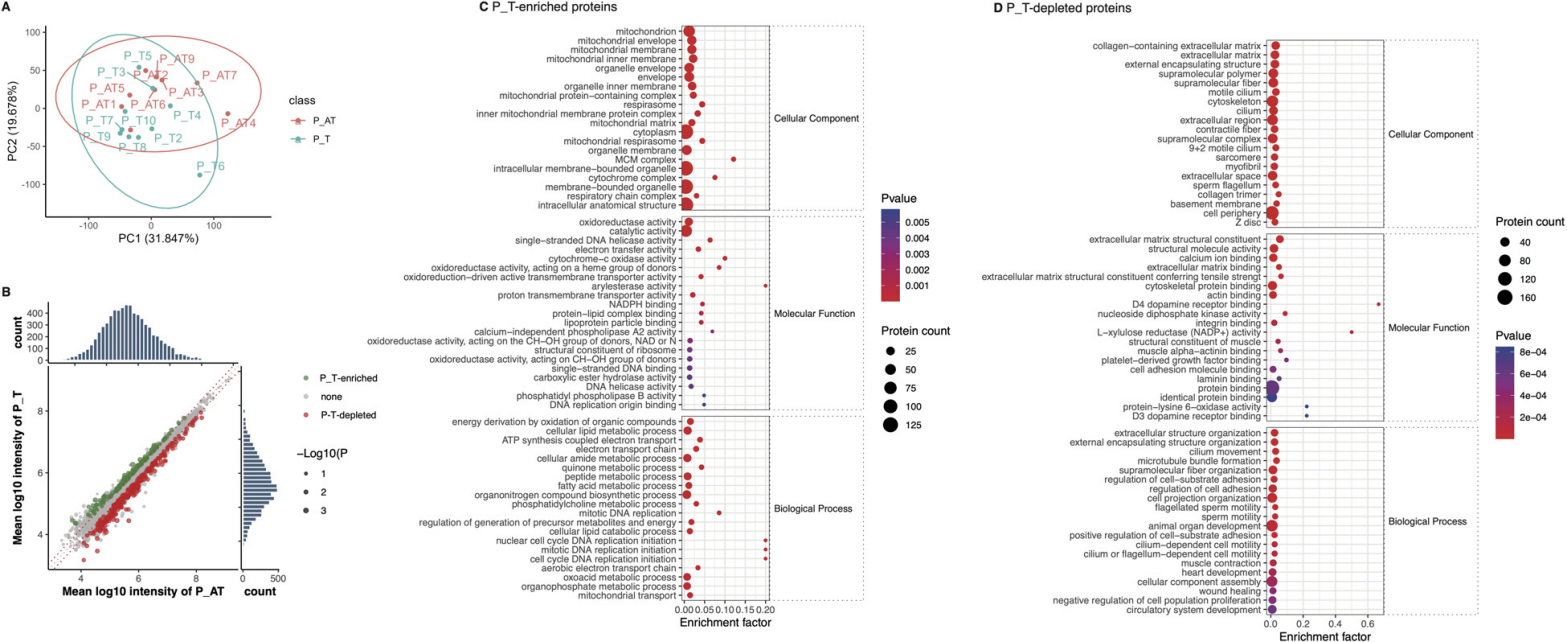
Differences of ovary protein expression between P_T and P_AT mice following therapies. **(A)** The separation of ovarian tissue samples between P_T(n=10) and P_AT(n=9) mice, according to profiling of identified proteins. **(B)** Differential expression of protein between P_T and P_AT mice. Green points represent P_T-enriched proteins, while red points represent P_T-depleted proteins. **(C)** Functional enrichment for P_T-enriched proteins. **(D)** Functional enrichment for P_T-depleted proteins.

We identified a range of 5,959 to 7,159 proteins within the ovarian tissues of 19 mice (**Supplementary Figure S2A**, **Supplementary Table S2**). Principal Coordinate Analysis (PCoA) indicated separation of ovarian tissue samples according to the normalized abundance of the identified proteins (**Figure 4A**). We then found that 152 proteins exhibited increased expression levels, while 270 proteins displayed decreased levels in P_T mice compared to the control group (**Figure 4B**).

Further functional enrichment analysis indicated that the 152 P_T-accumulated proteins were predominantly enriched in biological processes related to DNA replication initiation and mitotic DNA replication (**Figure 4C**). Additionally, these proteins were also enriched in metabolic processes involving quinone and electron transport (**Figure 4C**). At the molecular function level, we observed enrichment of enzyme activities associated with oxidoreduction and DNA replication (**Figure 4C**). Consistent with these findings, KEGG-based annotations further corroborated the enrichment of oxidative phosphorylation, DNA replication, and cell cycle-related functions among the P_T-accumulated proteins (**Supplementary Figure S2B**).

Conversely, the 270 P_T-depleted proteins were primarily enriched in biological processes related to cell adhesion and motility. The molecular functions of these depleted proteins were characterized by their binding capabilities to multiple molecules, including proteins and laminins (**Figure 4D**). In line with these observations, KEGG-based annotations indicated the enrichment of cell adhesion molecules and tight junction-associated proteins among the P_T-depleted proteins (**Supplementary Figure S2C**). These findings provide insights into the specific molecular mechanisms underlying the differential protein expression patterns observed in P_T mice.

## Discussion

The fertility of reproductive-aged women is significantly compromised by diminished ovarian function, which can stem from a multitude of disorders, including POI and primary ovarian failure (Carson and Kallen, 2021). Despite the widespread use of HRT, it falls short in restoring ovarian function to normal levels (Committee on Gynecologic Practice, 2017). TCM has been extensively employed in the management of ovarian dysfunction (Peng, 2015; Zhou et al., 2022; Tan et al., 2018; Duan and Cheng, 2021), yet the underlying mechanisms remain elusive. The present study underscores the pivotal role of GM in mediating the therapeutic efficacy of Gui-Shen-Wan in restoring ovarian function.

In our investigation, we found superior therapeutic outcomes with Gui-Shen-Wan in mice possessing intact GM compared to those with disrupted GM. This finding partially aligns with previous studies that emphasize the crucial role of GM in enhancing the beneficial effects of TCM (Zou et al., 2022; Lee et al., 2020; Gou et al., 2023; Li et al., 2023; Liu et al., 2023; Chen et al., 2023; Ammar et al., 2023; Huang et al., 2022; Cai et al., 2022; Zhou et al., 2022). Specifically, TCM can modulate GM compositions and even functional communities, thereby influencing phenotypic outcomes. For example, ginseng polysaccharides potentiated antitumor effects by altering GM metabolism (Huang et al., 2022). Similarly, Zuo-Jin-Wan exerted therapeutic effects against ulcerative colitis through modulating GM compositions and metabolism (Cai et al., 2022), while San-Wu-Huang-Qin decoction targets GM to mitigate tumorigenesis and mucosal barrier dysfunction (Zhou et al., 2022).

Another mechanism underpinning the role of GM in TCM therapy involves the GM-mediated metabolism of TCM components. A previous report demonstrated, the biotransformation of STW 5-II, a combination of six herbal extracts with clinically proven efficacy in functional dyspepsia (FD) and irritable bowel syndrome (IBS) (Ammar et al., 2023). During incubation with fecal microbiota, the levels of 110 constituents of STW 5-II varied, with 62 being metabolized into various metabolites, including compounds with established anti-inflammatory properties.

The correlation between GM and reproductive disorders, such as polycystic ovary syndrome, offers partial insight into the observed differences in ovarian function restoration between P_T and P_AT mouse models (Qi et al., 2019; Liang et al., 2021; Lüll et al., 2021). However, there is a notable gap in studies investigating the specific role of GM in mediating the beneficial effects of Gui-Shen-Wan. GM modulated estrogen levels through the secretion of β-glucuronidase (gmGUS), which converts conjugated estrogen into its deconjugated, bioactive form, enhancing its binding to estrogen receptors (Chadchan et al., 2022). Consequently, GM dysbiosis-associated impairments in gmGUS activity led to abnormal circulating estrogen levels, contributing to pathologies such as obesity and endometriosis (Mahboobifard et al., 2022; Alencar et al., 2021; Bulun et al., 2019). Furthermore, short-chain fatty acids (SCFAs) derived from GM influenced sex-steroid hormone levels. For instance, butyrate regulated the synthesis of progesterone (P4) and estradiol (E2) in porcine granulosa cells (PGCs) via the cAMP signaling pathway (Lu et al., 2017). Another study suggested that GM-derived butyrate contributed to nonalcoholic fatty liver disease in premenopausal women due to estrogen deficiency (Liu et al., 2022).

DNA replication is a fundamental process underpinning ovarian function and development (Tiosano et al., 2019). In this study, we observed that P_T mice exhibited enrichment of proteins associated with DNA replication and cell cycle regulation. Notably, the Mcm complex, a key player in DNA replication initiation (Nitani et al., 2008), was upregulated, with Mcm4 specifically contributing to accelerated cell proliferation (Tan et al., 2023). Additionally, P_T mice had enrichment of proteins related to oxidative phosphorylation, a vital process in follicular development that encompasses the mitochondrial electron transfer chain (Zhang et al., 2023). These findings suggest that the therapeutic effects of Gui-Shen-Wan on ovarian function may involve modulation of DNA replication and mitochondrial function, potentially mediated by GM.

Proteins depleted in P_T mice, including collagen and laminin, are primarily associated with cell adhesion and tight junction downregulation. This observation partially aligns with previous research highlighting the pivotal roles of cell adhesion and motility in ovarian development. Prior studies have shown that an age-related increase in collagen can lead to declined ovarian function, adversely affecting follicle development and oocyte quality (Amargant et al., 2020). Additionally, specific laminin isoforms have been implicated in malignant diseases, with laminin-332 playing a crucial role in the development of squamous cell carcinoma tumors (Patarroyo et al., 2002; Tran et al., 2008).

Though this study provides insights into the potential role of GM in mediating the therapeutic effects of Gui-Shen-Wan, several limitations should be acknowledged. Firstly, pathology assessment of ovarian tissues was not performed for control mice, and serum hormone levels were not measured across different experimental groups. Nevertheless, the observed disruption of menstruation in model mice compared to controls suggests cyclophosphamide-induced impairment of ovarian function. Given the study’s focus on elucidating the influence of GM on TCM therapeutic effects in model mice, the absence of pathological examination in controls does not detract from our primary findings. However, future studies incorporating hormone profiling, pathology detection, and proteome analysis of ovarian tissues would strengthen the understanding of therapeutic differences between P_T and P_AT mice. Secondly, differences in GM compositions between P_T and P_AT mice were not analyzed in this study. Nonetheless, the disruptive effects of the utilized antibiotics combination on GM have been well-documented in various studies (Liu et al., 2023; Qi et al., 2019; Liang et al., 2022) mitigating the impact of this limitation on our conclusions. Lastly, untargeted metabolomics analysis could be employed to further investigate the intricate interactions between GM and TCM, providing a more comprehensive understanding of the underlying mechanisms. This approach would enable the identification of metabolic changes associated with GM modulation and the therapeutic effects of Gui-Shen-Wan, enhancing the robustness of our findings.

In conclusion, our findings underscore the pivotal role of GM in mediating the beneficial effects of Gui-Shen-Wan in restoring ovarian function. Furthermore, we have delved into the association between GM and ovarian protein expression, providing valuable insights. This study offers crucial evidence that contributes to the understanding of the underlying mechanisms through which Gui-Shen-Wan exerts its therapeutic effects on diminished ovarian function. Such knowledge can potentially pave the way for novel therapeutic strategies targeting GM to improve ovarian health and fertility outcomes.

## Data Availability

The original contributions presented in the study are publicly available. This data can be found here: ProteomeXchange Consortium (https://proteomecentral.proteomexchange.org) via the dataset identifier PXD058216.
