# Supplementary figures and images for: The depletion of gut microbiome impairs the beneficial effect of Gui-Shen-Wan in restoring mice ovarian function and associated protein expression of ovarian tissues
Source: Front Cell Infect Microbiol. 2024 Nov 27;14:1505958. doi: 10.3389/fcimb.2024.1505958 (PMC11632464; doi:10.3389/fcimb.2024.1505958)

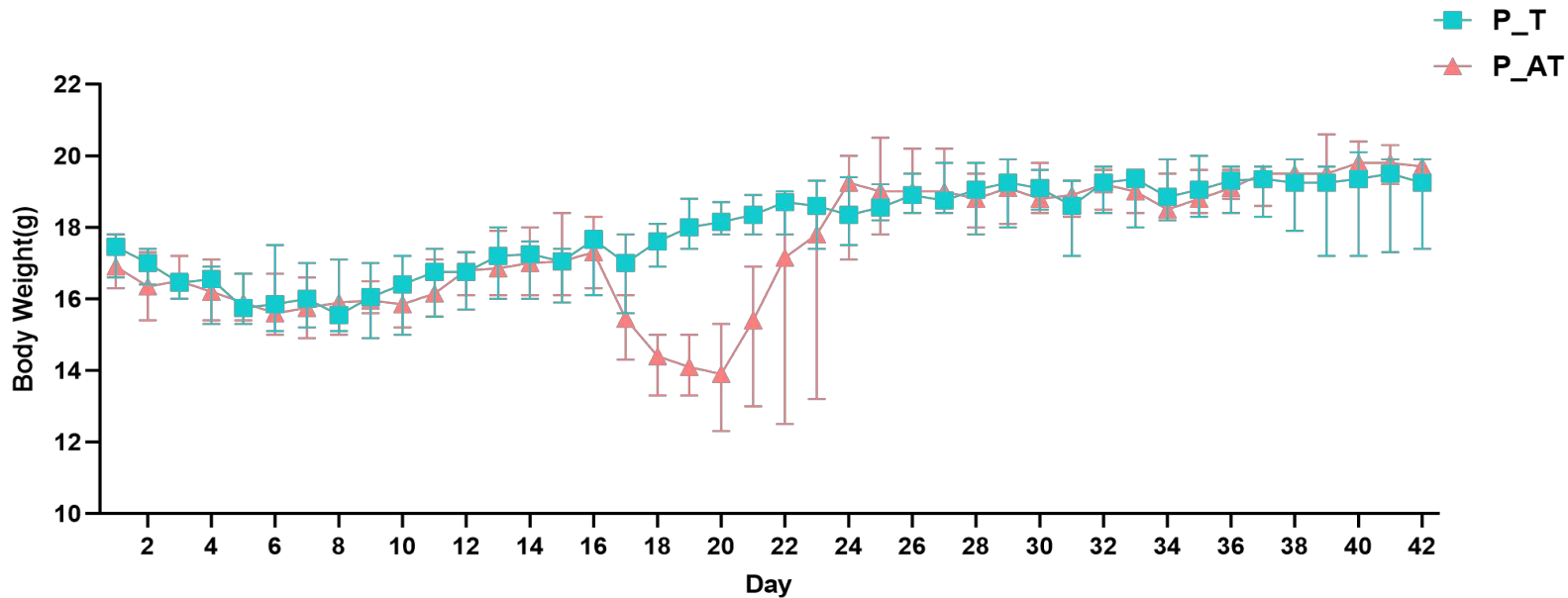

**Fig. S1** Differences of body weight between P\_T and P\_AT mice during the experiment.

Supplement: Supplementary file 1 [file DataSheet1.pdf]
