# Supplementary material for: The depletion of gut microbiome impairs the beneficial effect of Gui-Shen-Wan in restoring mice ovarian function and associated protein expression of ovarian tissues
Source: Front Cell Infect Microbiol. 2024 Nov 27;14:1505958. doi: 10.3389/fcimb.2024.1505958 (PMC11632464; doi:10.3389/fcimb.2024.1505958)
Supplement: Supplementary file 2 [file DataSheet2.pdf]

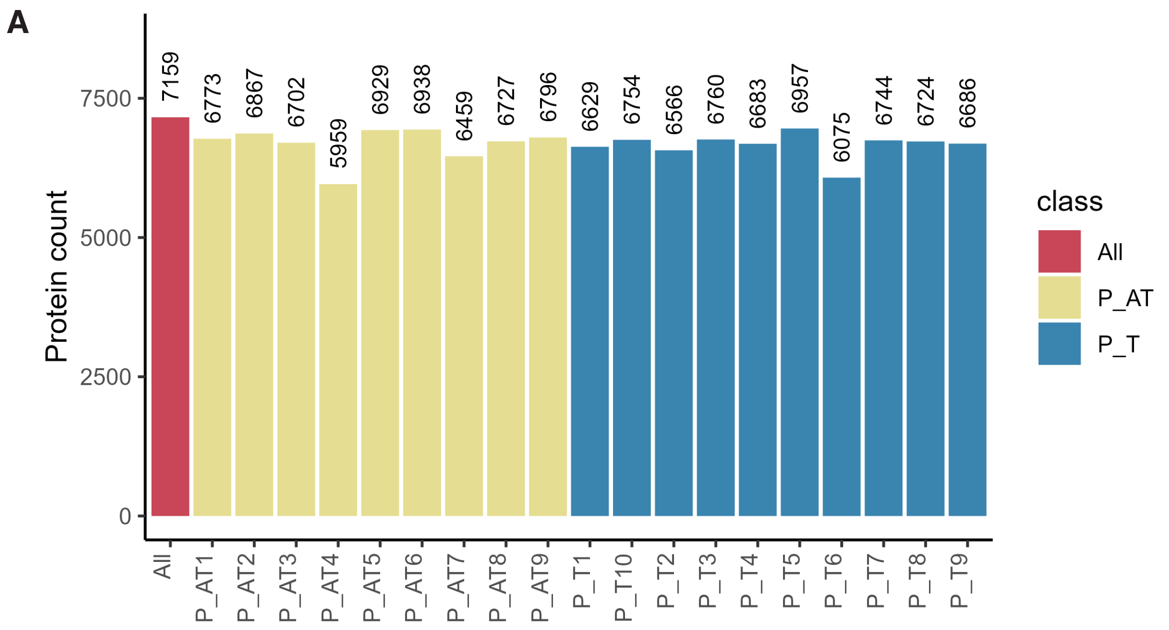


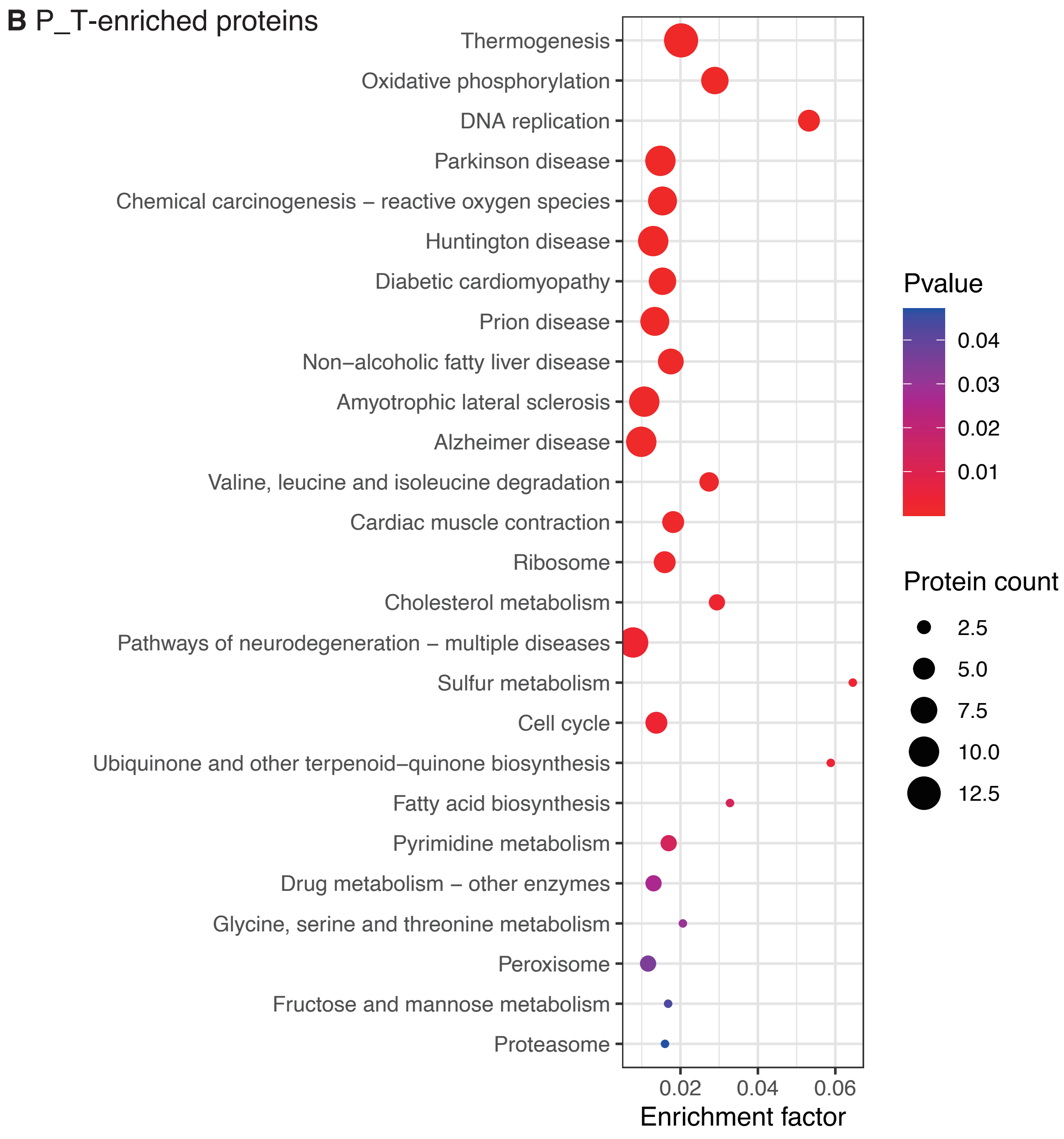


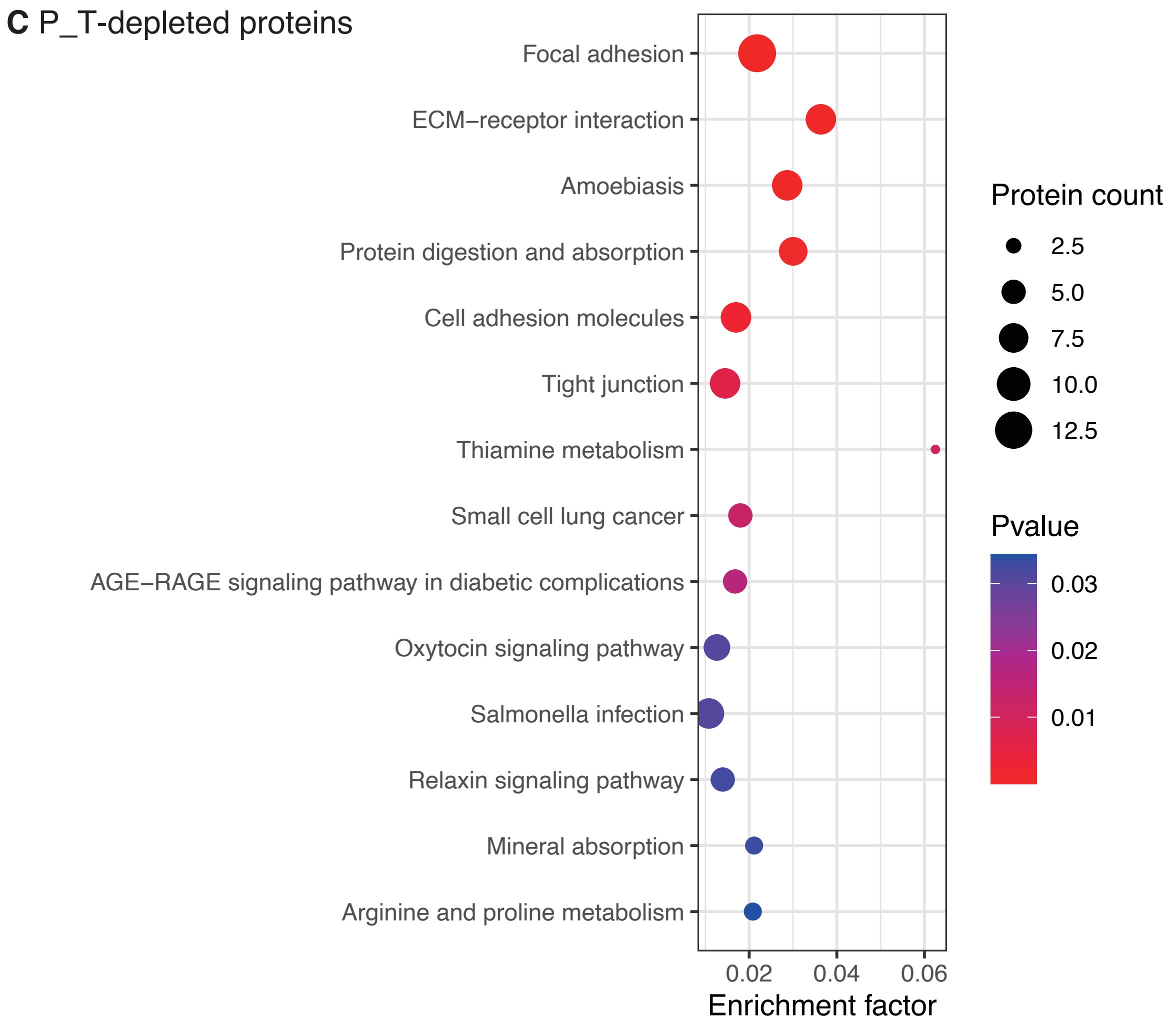


**Fig. S2** Additional results for protein expression of ovarian tissues for P_T and P_AT mice. **A**. The number of identified proteins for each sample. **B**. Functional enrichment of P_T-enriched proteins based on the KEGG database. **C**. Functional enrichement of P_T-depleted proteins via the KEGG database
