# Supplementary material for: The depletion of gut microbiome impairs the beneficial effect of Gui-Shen-Wan in restoring mice ovarian function and associated protein expression of ovarian tissues
Source: Front Cell Infect Microbiol. 2024 Nov 27;14:1505958. doi: 10.3389/fcimb.2024.1505958 (PMC11632464; doi:10.3389/fcimb.2024.1505958)
Supplement: Supplementary file 3 [file Table1.docx]

| Group | No. | Number of normal follicles |
| --- | --- | --- |
| P_T | 1 | 19 |
|  | 2 | 19 |
|  | 3 | 15 |
|  | 4 | 15 |
|  | 5 | 16 |
|  | 6 | 9 |
|  | 7 | 8 |
|  | 8 | 11 |
|  | 9 | 7 |
|  | 10 | 17 |
| P_AT | 1 | 5 |
|  | 2 | 10 |
|  | 3 | 15 |
|  | 4 | 12 |
|  | 5 | 7 |
|  | 6 | / |
|  | 7 | 4 |
|  | 8 | 4 |
|  | 9 | 11 |
